# Validation of the Turkish version of the Phobic Stimuli Response Scales

**DOI:** 10.1186/s41155-026-00380-7

**Published:** 2026-01-30

**Authors:** Esef Ercüment Yerlikaya, Meryem Dedeler

**Affiliations:** Faculty of Arts and Sciences, Department of Psychology, Çukurova University, Adana, Sarıçam, 01250 Türkiye

**Keywords:** Fear, Phobias, Phobic stimuli, Validity, Reliability

## Abstract

**Background:**

The current literature lacks measurement tools that cover all subtypes of specific phobias.

**Objective:**

This study aimed to adapt the Phobic Stimuli Response Scales (PSRS) into Turkish and evaluate its psychometric properties.

**Methods:**

399 university students (308 females, 77.2%; 91 males, 22.8%) aged between 18 and 53 years (*M* = 21.80, *SD* = 3.93) filled out the PSRS, Fear Survey Schedule-II, and Depression, Anxiety, Stress Scale (DASS-21) online. 108 participants took part in the test-retest study 15 days after the first application.

**Results:**

According to confirmatory factor analysis, model fit indices (CFI = 0.97, TLI = 0.97, RMSEA = 0.07, and SRMR = 0.07) showed that the Turkish form of the scale consisted of 46 items and five factors (Blood-Injection, Bodily Harm, Social, Animal, and Physical Confinement) as in the original. Its correlations with other scales showed its convergent and discriminant validity. The reliability analyses indicate that the scale demonstrates internal consistency (*α* = 0.78 for Bodily Harm, 0.85 for Physical Confinement, 0.88 for Social, 0.86 for Animal, 0.90 for Blood-Injection, and 0.94 for the total score) and test-retest reliability.

**Conclusions:**

The findings suggest that the Turkish PSRS is a reliable and valid instrument for assessing responses to various fears in research and practical applications.

**Supplementary Information:**

The online version contains supplementary material available at 10.1186/s41155-026-00380-7.

## Introduction

American Psychological Association (APA) Dictionary of Psychology defines phobia as “a persistent and irrational fear of a specific situation, object, or activity (e.g., heights, dogs, water, blood, driving, flying), which is consequently either strenuously avoided or endured with marked distress” (VandenBos, 2015, p. 792). Fear in this definition is defined as “a basic, intense emotion aroused by the detection of imminent threat, involving an immediate alarm reaction that mobilizes the organism by triggering a set of physiological changes” (VandenBos, 2015, p. 413). Notably, specific phobia is defined as an anxiety disorder—previously known as simple phobia—characterized by an intense and persistent fear of a particular object, activity, or situation (e.g., dogs, blood, flying, heights). “The fear is traditionally defined as excessive or unreasonable and is invariably triggered by the presence or anticipation of the feared object or situation, which is either avoided or endured with marked anxiety or distress” (VandenBos, 2015, p. 1014).

The Diagnostic and Statistical Manual of Mental Disorders Fourth Edition Text Revision (DSM-IV-TR) classifies specific phobias under Anxiety Disorders, alongside other conditions such as panic disorder (with or without agoraphobia), agoraphobia (without a history of panic disorder), social phobia, obsessive-compulsive disorder, post-traumatic stress disorder, and generalized anxiety disorder (American Psychiatric Association [APA], 2000). DSM-IV-TR specifies five main specific phobia types: animal type, natural environment type, blood-injection-injury type, situational type, and other type (APA, 2000). In the Diagnostic and Statistical Manual of Mental Disorders, Fifth Edition (DSM-5), specific phobias are classified under Anxiety Disorders, along with separation anxiety disorder, selective mutism, social anxiety disorder (social phobia), panic disorder, agoraphobia, generalized anxiety disorder, substance-medication induced anxiety disorder, and anxiety disorder due to another medical condition (APA, 2013). Similar to DSM-IV-TR, in DSM-5, specific phobias have five types: animal type (e.g., spiders, snakes, dogs, etc.), natural environment type (e.g., heights, tornadoes, water, etc.), blood-injection-injury type (e.g., needles, medical procedures), situational type (e.g., flying on an airplane, elevators, enclosed spaces), and other type (phobias that do not fit into the previous four categories) (APA, 2013). The DSM-5 maintains these subtypes but removes the earlier requirement that fear must be excessive or unreasonable for diagnosis. Instead, it specifies that fear should be disproportionate to the actual threat posed by the object, situation, or its context (VandenBos, 2015). Specific phobia is a common and impairing anxiety disorder, with prevalence estimates ranging from 2% to 12.5% in epidemiological studies (Kessler et al., 2005a, b; Stinson et al., 2007) and lifetime rates reported between 7% and 13% in North American samples (Eaton et al., 2018). The global lifetime prevalence has been similarly estimated at approximately 7.4% (Wardenaar et al., 2017). The disorder typically emerges early in life, often follows a chronic course in the absence of treatment—with 17-month natural remission rates as low as 19% (Trumpf et al., 2010)—and is associated with substantial social, occupational, and physical impairment, including avoidance of important medical procedures (Wolitzky-Taylor et al., 2008). Moreover, comorbidity is common: more than 60% of individuals with specific phobia experience additional mental health disorders throughout their lifetime, particularly other anxiety and mood disorders (Wardenaar et al., 2017). These impairments are accompanied by lower self-efficacy, elevated stress, reduced decision-making capacity, and diminished quality of life (Essau et al., 2014; Zhang et al., 2020).

Although exposure-based interventions are highly effective and represent the gold standard treatment for specific phobia (Eaton et al., 2018; Grös & Antony, 2006), accurate assessment is essential for guiding intervention planning and improving treatment outcomes. Misdiagnosis or under-detection can lead to inappropriate treatment choices, poorer symptom reduction, and higher attrition rates (Cook & Décary, 2020; Jensen-Doss & Weisz, 2008). Despite the high prevalence and clinical burden associated with the condition, specific phobia remains considerably under-recognized and under-treated (Bandelow & Michaelis, 2015; Eaton et al., 2018), underscoring the need for reliable and valid assessment tools.

Although clinician-administered interviews such as the Anxiety and Related Disorders Interview Schedule for DSM-5 (ADIS-5; Brown & Barlow, 2014) and the Structured Clinical Interview for DSM-5 (SCID; First et al., 2015) are considered the gold standard for diagnosing specific phobias, their cost and administration time limit their use as screening tools. Self-report measures provide a more efficient alternative; however, widely used instruments such as the Fear Survey Schedule-II (Geer, 1965) and the Fear Survey Schedule-III (Wolpe & Lang, 1977) have shown notable limitations. These measures primarily list fear-provoking stimuli and therefore offer little information about the cognitive or emotional processes underlying fear. Research has also shown that the FSS scales struggle to reliably distinguish individuals with and without specific phobias (Beck et al., 1998), show inconsistent factor structures, and inadequately capture DSM-defined subtypes, particularly natural environment fears (Muris et al., 1999).

To address these shortcomings, Cutshall and Watson (2004) developed a scale assessing the cognitive and emotional aspects of fear responses. They intentionally avoided labeling it as a “phobia” since their data were collected from an undergraduate sample. However, they preferred to name the scale the Phobic Stimuli Response Scales (PSRS), recognizing that nonclinical samples still experience fear and that such data can provide valuable insights for clinical populations (Cutshall & Watson, 2004). Additionally, they used full sentences to describe emotional responses to a given stimulus, as explicit wording helps minimize individual differences in item interpretation and provides a clearer understanding of various emotional reactions (Cutshall & Watson, 2004). Unlike earlier instruments, the PSRS evaluates not only the presence of feared stimuli but also the cognitive and affective responses associated with them, providing a more nuanced assessment of fear. Importantly, the PSRS item pool was designed to represent all DSM-specific phobia subtypes, allowing the first direct test of this classification. Factor analytic studies identified five stable dimensions—Animal, Blood-Injection, Physical Confinement, Bodily Harm, and Social—three of which map directly onto specific phobia subtypes, while the Bodily Harm factor broadens the assessment of natural environment fears beyond traditional definitions.

Overall, the PSRS offers a more comprehensive, psychometrically robust, and clinically meaningful alternative to traditional fear survey schedules, making it well-suited for screening, classification, and monitoring of specific phobia symptoms.

Since its development, the PSRS (Cutshall & Watson, 2004) has been widely applied in research, both as a full measure and through its individual subscales. Several studies have used the full scale to examine fear, anxiety, and related constructs (e.g., MacLeod et al., 2022; Moliné et al., 2018; Naragon-Gainey & Watson, 2011; Ovanessian et al., 2019; Stanton & Watson, 2015; Thibodeau et al., 2015; Watson et al., 2012, 2017). At the same time, the PSRS subscales—including Animal, Blood-Injection, Physical Confinement, Bodily Harm, and Social—have been selectively used to investigate domain-specific fears, social anxiety, phobic tendencies, and associations with broader psychopathology (e.g., Carter & Wu, 2010; Koffel & Watson, 2009; Levin-Aspenson et al., 2021; Longley et al., 2006; Naragon-Gainey et al., 2009; Stasik-O’Brien et al., 2021). Together, these studies demonstrate that the PSRS has remained a versatile and frequently utilized instrument for assessing diverse phobic stimuli and related psychological processes.

Although several self-report instruments exist in Turkish to assess phobic symptoms, these measures largely target single fear domains, such as agoraphobia (Aydın et al., 2017), blood–injury phobia (Ak et al., 2014), and social phobia (Cengiz et al., 2018), each evaluated through separate scales. Consequently, the field lacks a comprehensive tool capable of assessing the broad spectrum of fear responses across multiple specific phobia subtypes within a single framework. The only multidimensional instrument currently available—the DSM-5 Severity Measure for Specific Phobia (Adult)—provides a brief 10-item assessment of anxiety, fear, and avoidance related to five situations or objects, rated according to symptom frequency over the past week (Craske et al., 2013; Turkish version by Öztekin et al., 2017). While clinically useful, this scale evaluates only symptom severity and does not capture the cognitive and emotional components of phobic responses or the distinct dimensions underlying different phobia types. In contrast, the PSRS offers a theoretically grounded, multidimensional assessment that includes five fear domains and evaluates individuals’ cognitive–emotional reactions to phobic stimuli. Therefore, adapting the PSRS into Turkish is essential to address the current gap in the literature and to provide researchers and clinicians with a more comprehensive and psychometrically robust instrument for the assessment of specific phobias.

Hence, this study aimed to adapt the PSRS into Turkish and evaluate its psychometric properties in a nonclinical sample for several reasons: (1) The five subtypes of specific phobia have remained unchanged from the DSM-IV-TR to the DSM-5, ensuring that the scale remains relevant and comprehensive. (2) The use of full-sentence items enhances participants’ understanding of phobic stimuli. (3) While clinical interviews are valuable for diagnosing phobias, they are impractical for large-scale studies, necessitating a comprehensive measurement tool for assessing phobias and fears. (4) To the best of our knowledge, no such measurement tool currently exists in Turkish literature. Therefore, adapting the PSRS into Turkish and examining its psychometric properties is crucial. This adaptation will enable Turkish researchers interested in phobias and fears to conduct large-scale studies and facilitate the identification of specific phobias (rather than diagnosis) in clinical settings.

Accordingly, this study sought to adapt the PSRS into Turkish and assess its psychometric properties in a non-clinical sample of university students. It was hypothesized that the Turkish version of the PSRS would (1) maintain a multidimensional factor structure consistent with the original scale, (2) exhibit strong internal consistency and reliability, and (3) demonstrate adequate validity in assessing phobic responses.

## Methods

### Participants

The study included 399 university students (308 females, 77.2%; 91 males, 22.8%) aged between 18 and 53 years (*M* = 21.80, *SD* = 3.93), with 94% of them being 26 or younger. Regarding academic year distribution, 111 participants (27.8%) were in their first year, 74 (18.5%) in their second year, 104 (26.1%) in their third year, 92 (23.1%) in their fourth year, and 18 (4.5%) had been enrolled in university for five or more years.

### Instruments

#### Informed consent form

Before participating in the study, individuals were informed through an informed consent form that participation was voluntary, that they had to be university students aged 18 or older, that the study focused on fears, and that they could withdraw at any time.

#### Sociodemographic information form

This form, developed by the researchers, includes three questions about participants’ age, gender, and years of university enrollment.

#### The phobic stimuli response scales (PSRS)

 Developed by Cutshall and Watson (2004), the PSRS is a 46-item scale designed to assess the cognitive and emotional aspects of fear. It is rated on a 4-point Likert-type scale (1 = Strongly Disagree, 4 = Strongly Agree) and comprises five sub-dimensions: Social, Animal, Physical Confinement, Bodily Harm, and Blood-Injection. The internal consistency coefficients were reported as 0.80 for Bodily Harm, 0.81 for Physical Confinement, 0.85 for Social, 0.86 for Animal, 0.87 for Blood-Injection, and 0.88 for the overall scale.

#### Fear survey schedule II (FSS-II)

The Fear Survey Schedule, Second Edition (FSS-II), developed by Geer (1965), assesses the intensity of fear related to various situations and stimuli. The scale consists of 51 items rated on a 7-point Likert scale (1 = None, 7 = Terror). The Cronbach’s alpha internal consistency coefficient was reported as 0.94 for the original scale and 0.95 in the present study. Although a Turkish adaptation has not yet been conducted, the scale was used in this study after being translated into Turkish by the authors.

#### Depression, anxiety, stress scale (DASS-21)

The DASS-21, developed by Lovibond and Lovibond (1995), measures depression, anxiety, and stress. It consists of 21 items rated on a 4-point Likert-type scale (0 = Did not apply to me at all; 3 = Applied to me very much or most of the time). In the Turkish version, responses range from 0 (Never) to 3 (Always), with Cronbach’s alpha values reported as 0.87 for depression, 0.85 for anxiety, and 0.81 for stress (Sarıçam, 2018; Sarıçam et al., 2015). In the present study, Cronbach’s alpha coefficients were 0.87, 0.82, and 0.80, respectively.

### Procedure

Before conducting the study, permission was obtained from the original authors (corresponding author Cutshall) for the Turkish adaptation of the PSRS. After receiving the necessary permission, the translation process began. During the translation process, the authors followed the guidelines (e.g., ITC Guidelines for Translating and Adapting Tests, 2017) regarding scale adaptation processes. Firstly, the scale was translated into Turkish separately by the researchers, and then the consistency between the translations was examined to determine the final form of the Turkish scale. Following this, a back-translation of the Turkish scale into English was conducted by an independent field expert to ensure compatibility with the original version. Subsequently, the Turkish scale was applied to a pilot sample of five individuals to examine its comprehensibility. Following this process, data collection commenced after obtaining ethical approval from Çukurova University’s Ethics Committee (Date: 22 July 2022; Number: 124/52).

Participants were recruited through convenience sampling, with the inclusion criteria requiring them to be university students aged 18 or older. The researchers disseminated the study poster and participation link via class groups and personal social media platforms and encouraged university students who completed the scales to forward the link to another participant. Data were collected via Google Forms between February and July 2024. To assess test-retest reliability, volunteer participants were asked to provide their email addresses and nicknames at the end of the scale battery. Those who consented were contacted via email 15 days later and received a link to complete the PSRS again.

### Data analysis

All statistical analyses were performed using JASP software (JASP Team, 2023). To provide validity evidence based on the internal structure, a confirmatory factor analysis was conducted. Convergent (the relationship between the PSRS total score and FSS-II total score) and discriminant (the relationship of DASS-21 subscales with the PSRS subscales and the PSRS total score) validity, as well as test–retest reliability, were examined using Pearson’s product-moment correlation analysis. Additionally, the intraclass correlation coefficient was calculated to further evaluate test-retest reliability. For reliability assessment, Cronbach’s alpha coefficients, along with corrected item–total and item–subscale correlations, were computed. Finally, descriptive statistics, including means and standard deviations, were calculated to examine item-level characteristics.

## Results

Before conducting validity and reliability analyses, the internal consistency coefficient was calculated for all items, yielding a value of 0.94. Next, item-total correlations were examined, ranging from 0.21 (Item 35: “I am not shy around people I’ve just met.” [reverse-coded]) to 0.62 (Item 28: “I get nervous when people are watching me.”).

**Fig. 1:**
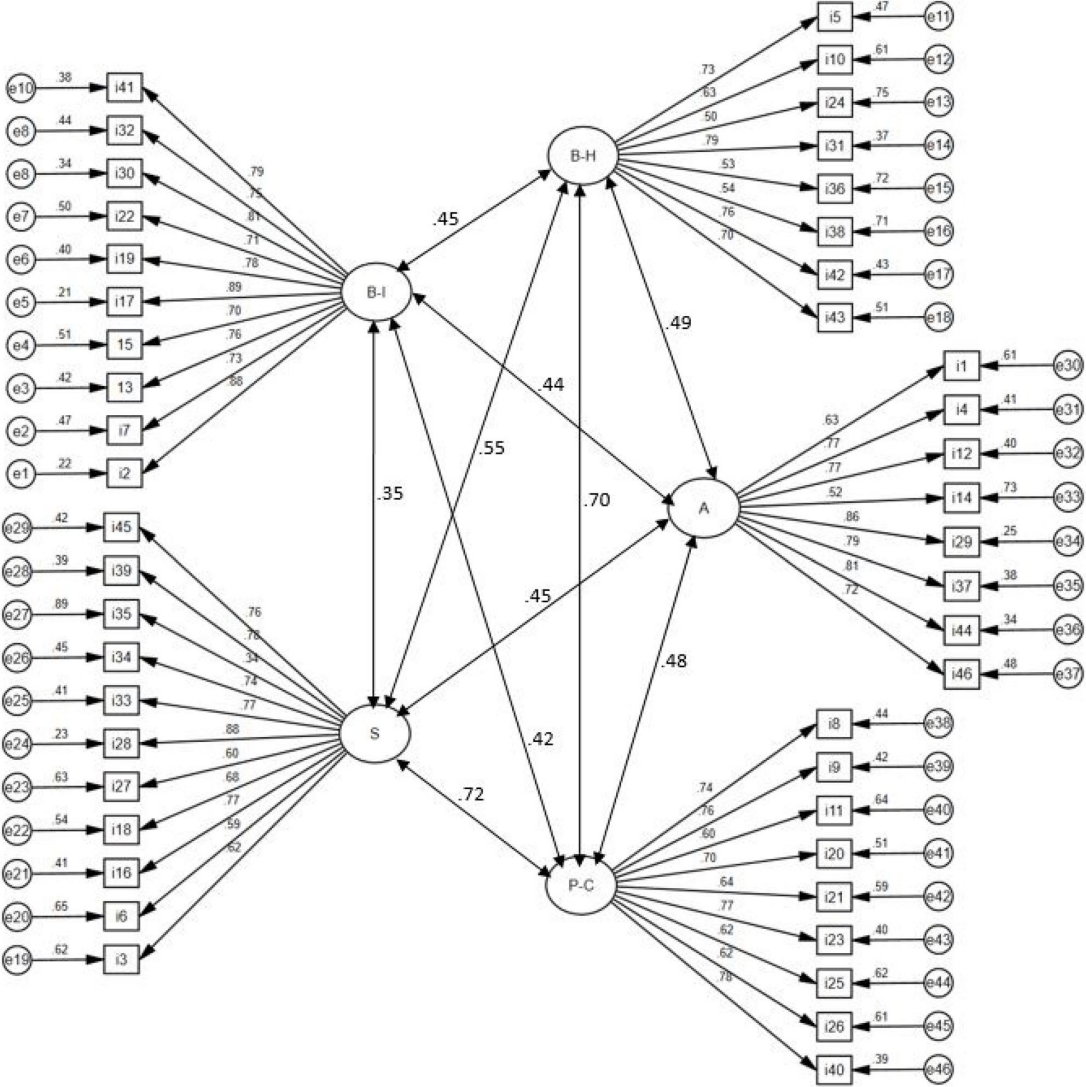
Path Diagram for Confirmatory Factor Analysis. Note. S = Social, A = Animal, B-I = Blood-Injection, B-H = Bodily Harm, and P-C = Physical Confinement

To assess the validity evidence based on the internal structure of the scale, confirmatory factor analysis (CFA) was performed using the Diagonally Weighted Least Squares (DWLS) estimation method. The analysis tested whether the original five-factor structure (Blood-Injection, Bodily Harm, Social, Animal, and Physical Confinement) applied to the Turkish context. Model fit was evaluated based on the Comparative Fit Index (CFI), Tucker-Lewis Index (TLI), Root Mean Square Error of Approximation (RMSEA), and Standardized Root Mean Square Residual (SRMR). No modification was used in the CFA. The fit indices indicated adequate support for the five-factor structure, with factor loadings ranging from 0.50 to 0.89, except for item 35 (*λ* = 0.34, though still significant). Model fit indices are presented in Table 1, factor loadings in Table 2, and the path diagram in Fig. 1.

**Table 1:** Confirmatory factor analysis results of the PSRS

Index	Value
X²	2847.27
df	979
CFI	0.97
TLI	0.97
RMSEA	0.07
SRMR	0.07

Note. *CFI* Comparative Fit Index, *TLI* Tucker-Lewis Index, *RMSEA* Root Mean Square Error of Approximation, *SRMR* Standardized Root Mean Square Residual

**Table 2:** Item statistics and factor loadings of the PSRS

Items	*M*	*SD*	Corrected Item-Subscale	Corrected Item-Total	Factor Loading
**Factor **					
**I. Blood-Injection: 10 items**					
PSRS 2	2.27	1.07	0.80	0.53	0.88
PSRS 7	2.03	0.89	0.60	0.46	0.73
PSRS 13	2.22	1.02	0.59	0.45	0.76
PSRS 15	2.46	1.00	0.58	0.50	0.70
PSRS 17	1.72	0.81	0.78	0.46	0.89
PSRS 19	1.95	0.96	0.72	0.44	0.78
PSRS 22	2.27	0.96	0.58	0.49	0.71
PSRS 30	2.25	1.01	0.67	0.47	0.81
PSRS 32	2.09	0.96	0.67	0.42	0.75
PSRS 41	2.47	1.02	0.62	0.55	0.79
**II. Bodily Harm: 8 items**					
PSRS 5	2.21	1.01	0.51	0.45	0.73
PSRS 10	2.95	0.88	0.56	0.44	0.63
PSRS 24	2.62	1.06	0.29	0.36	0.50
PSRS 31	3.06	0.91	0.55	0.58	0.80
PSRS 36	2.44	0.97	0.36	0.38	0.53
PSRS 38	2.49	1.00	0.46	0.36	0.54
PSRS 42	2.36	0.98	0.58	0.50	0.76
PSRS 43	2.69	0.95	0.61	0.49	0.70
**III. Social: 11 items**					
PSRS 3	2.74	0.88	0.54	0.44	0.62
PSRS 6	2.66	0.90	0.53	0.44	0.59
PSRS 16	2.95	0.80	0.65	0.53	0.77
PSRS 18	2.70	0.95	0.61	0.48	0.68
PSRS 27	2.39	0.86	0.52	0.42	0.60
PSRS 28	2.86	0.82	0.76	0.62	0.88
PSRS 33	2.60	0.91	0.60	0.56	0.77
PSRS 34	2.66	0.82	0.66	0.52	0.74
PSRS 35^a^	2.67	0.90	0.37	0.21	0.34
PSRS 39	2.64	0.81	0.63	0.60	0.78
PSRS 45	2.46	0.86	0.60	0.54	0.76
**IV. Animal: 8 items**					
PSRS 1	2.76	0.89	0.60	0.39	0.63
PSRS 4	3.17	0.85	0.64	0.52	0.77
PSRS 12	3.10	0.98	0.64	0.48	0.77
PSRS 14^a^	3.53	0.86	0.37	0.28	0.52
PSRS 29	3.25	0.89	0.69	0.55	0.86
PSRS 37	3.23	0.87	0.69	0.47	0.79
PSRS 44	3.44	0.84	0.66	0.50	0.82
PSRS 46	3.16	0.88	0.57	0.48	0.72
**V. Physical Confinement: 9 items**					
PSRS 8	2.80	0.96	0.63	0.56	0.75
PSRS 9	2.80	1.00	0.63	0.59	0.76
PSRS 11	3.09	0.87	0.53	0.46	0.60
PSRS 20	3.32	0.92	0.48	0.52	0.70
PSRS 21	2.22	0.89	0.52	0.49	0.64
PSRS 23	2.78	0.89	0.71	0.58	0.77
PSRS 25	2.73	0.88	0.57	0.39	0.62
PSRS 26	3.01	0.84	0.53	0.46	0.62
PSRS 40	2.94	0.92	0.58	0.61	0.78

Note. ^a^ Reverse-coded items

After the CFA results confirmed the validity of the five-factor, 46-item structure, the scale’s convergent and discriminant validity were examined. Convergent validity was assessed by examining the correlations between the PSRS total score and the FSS-II total score. For discriminant validity, correlations between the Depression, Anxiety, and Stress Scale-21 (DASS-21) subscale scores and the PSRS subscale and total scores were assessed. Additionally, the intercorrelations among the PSRS subscales and their relationships with depression, anxiety, and stress were analyzed. The findings are presented in Table 3.

**Table 3:** Convergent and discriminant validity of the PSRS

	1	2	3	4	5	6	7	8	9	10
1. PSRS Total Score	-									
2. Blood-Injection	0.70*	-								
3. Bodily Harm	0.76*	0.39*	-							
4. Social	0.77*	0.31*	0.48*	-						
5. Animal	0.69*	0.39*	0.43*	0.41*	-					
6. Physical Confinement	0.81*	0.38*	0.62*	0.64*	0.42*	-				
7. FSS-II Total Score	0.81*	0.48*	0.70*	0.67*	0.52*	0.67*	-			
8. Depression	0.33*	0.19*	0.17*	0.39*	0.16*	0.30*	0.34*	-		
9. Anxiety	0.44*	0.20*	0.35*	0.46*	0.23*	0.41*	0.48*	0.63*	-	
10. Stress	0.40*	0.20*	0.28*	0.37*	0.28*	0.38*	0.41*	0.66*	0.68*	-

* *p* < 0.001

After assessing the validity of the scale, reliability analyses were conducted. First, Cronbach’s alpha was calculated to evaluate the internal consistency of the PSRS total score and its sub-dimensions. The internal consistency coefficients were 0.78 for Bodily Harm, 0.85 for Physical Confinement, 0.88 for Social, 0.86 for Animal, 0.90 for Blood-Injection, and 0.94 for the total score.

Secondly, to evaluate the test-retest reliability of the scale, data were collected for a second time following a 15-day interval from 108 participants (87 females, 21 males) who had agreed to participate in the test-retest study from the initial sample of 399. The correlation between the two administrations was examined to assess the stability of the scale over time. The mean and standard deviation values of the PSRS in the first and second administrations, along with their correlations, and the intraclass correlation coefficients (ICCs) are presented in Table 4.

**Table 4:** Test-retest reliability of the PSRS

Subscale	Time 1 Mean (SD)	Time 2 Mean (SD)	*r*	ICC	Cronbach’s α (Time 2)
PSRS TOTAL	123.81 (20.31)	124.06 (20.41)	0.94*	0.94	0.94
Blood-Injection	21.46 (6.89)	21.85 (6.98)	0.93*	0.93	0.91
Bodily Harm	20.73 (5.22)	20.91 (5.06)	0.90*	0.90	0.81
Social	29.84 (5.71)	30.00 (5.59)	0.87*	0.87	0.87
Animal	26.12 (4.54)	25.80 (4.49)	0.90*	0.90	0.84
Physical Confinement	25.65 (5.81)	25.50 (5.77)	0.88*	0.88	0.89

* *p* < 0.001, *N* = 108

## Discussion

The Phobic Stimuli Response Scales (PSRS; Cutshall & Watson, 2004) is a self-report measure designed to assess cognitive and emotional responses to five types of fears: social, animal, physical confinement, bodily harm, and blood-injection. This study aimed to adapt the PSRS into Turkish and evaluate its psychometric properties in a non-clinical sample of university students. It was hypothesized that the Turkish version of the PSRS would (1) maintain a multidimensional factor structure consistent with the original scale, (2) demonstrate strong internal consistency and reliability, and (3) show satisfactory validity in assessing phobic responses.

The first hypothesis was supported, as the findings indicated that the Turkish version of the PSRS exhibited a strong fit to the hypothesized factor structure. Model fit indices met the acceptable thresholds established in the literature (Byrne, 1994; Hu & Bentler, 1999; Schumacker & Lomax, 2004; Tucker & Lewis, 1973), with an X²/df ratio of 2.91 (< 3), CFI and TLI values of 0.97 (> 0.90), and RMSEA and SRMR values of 0.07 (< 0.08), all indicating a good model fit. Furthermore, the five distinct dimensions of phobic responses identified in the original scale were retained in the Turkish adaptation, suggesting that the factorial structure of the PSRS remains stable across cultures. These results indicate that the Turkish adaptation retains its multidimensional structure and accurately reflects different phobic response dimensions. This finding underscores the robustness of the PSRS in capturing a broad range of phobic responses and further validates its ability to differentiate between distinct fear categories. Upon analyzing the factor loadings, it was observed that all items, except for item 35 (“I am generally not shy around people I’ve just met.“), had standardized loadings above 0.50—a commonly accepted threshold for item retention in factor analysis (Hair et al., 2009). Although the loading remained statistically significant, its magnitude suggests that the item may not represent the underlying construct as strongly as the rest of the scale. Given that item 35 assesses social ease rather than a direct fear response, cultural interpretations of shyness, interpersonal comfort, or social desirability may influence how Turkish participants respond to this item. Future studies should therefore examine whether item 35 would benefit from revision to improve clarity or cultural relevance, or whether removing it altogether would enhance the scale’s factorial precision without compromising its theoretical scope. Replication in clinical samples and cognitive interviewing methods may also help clarify whether the item reflects a broader personality trait rather than a domain-specific phobic tendency. Nevertheless, the item was retained in the Turkish adaptation to maintain consistency with the original scale and to preserve the structural integrity of the full 46-item measure.

The second hypothesis was supported, as the Turkish version of the PSRS demonstrated strong convergent and discriminant validity. Convergent validity was assessed by examining the correlations between the PSRS total score and the FSS-II total score. The PSRS total score showed a strong positive correlation with the FSS-II total score (*r* = 0.81, *p* < 0.001), indicating that both scales measure closely related constructs of fear and phobic responses. This finding aligns with previous research in literature. For instance, MacLeod et al. (2022) reported a significant association between the PSRS total score and the FSS-II (*r* = 0.83, *p* < 0.001), further supporting the convergent validity of the PSRS.

Discriminant validity was evaluated by analyzing the intercorrelations among the PSRS subscales and their relationships with depression, anxiety, and stress. In line with Ashton et al. (2008), the PSRS subscales showed moderate intercorrelations (ranging from *r* = 0.31 to *r* = 0.64, *p* < 0.001), suggesting that while the fear domains are related, they remain distinct constructs. Additionally, as expected, the PSRS total and subscale scores exhibited lower correlations with measures of general distress, a pattern consistent with the findings of MacLeod et al. (2022). The highest correlation was found between PSRS total and anxiety (*r* = 0.44, *p* < 0.001), followed by stress (*r* = 0.40, *p* < 0.001), and depression (*r* = 0.33, *p* < 0.001). These findings indicate that although fear-related constructs may share some overlap with emotional distress, the PSRS primarily assesses phobic responses rather than general negative affect.

The third hypothesis was supported as the reliability analysis demonstrated that the Turkish version of the PSRS exhibits strong internal consistency. The Cronbach’s alpha coefficients for the subscales ranged from 0.78 (Bodily Harm) to 0.90 (Blood-Injection), while the total score showed excellent reliability (*α* = 0.94). These values indicate that the scale provides stable and reliable measurements across different fear domains. Notably, the high internal consistency of the total score suggests that the PSRS effectively captures a broad range of phobic responses. The reliability coefficients obtained in this study align well with those found in the original validation (Cutshall & Watson, 2004) and similar studies (e.g., Ashton et al., 2008; MacLeod et al., 2022), providing further evidence for the robustness and reliability of the Turkish adaptation. Furthermore, test-retest reliability analysis confirmed the scale’s temporal stability, indicating strong consistency between the first and second applications. The test-retest correlation coefficients (also the intraclass correlation coefficients) ranged from 0.87 to 0.93 for the subscales and 0.94 for the total score, demonstrating high reliability over time.

Beyond its psychometric strengths, the PSRS provides valuable insights into the classification of phobic responses. The original validation study suggested that some DSM-defined fear categories, such as natural environment phobias, might be more closely associated with bodily harm fears. Although the current study did not explicitly test this hypothesis, the findings indicate that future research could explore whether specific fear domains in the Turkish population align with existing classification systems or whether cultural factors influence their organization.

Furthermore, prior research suggests that phobic responses can be influenced by personality traits such as neuroticism, extraversion, and disgust sensitivity. While the current study did not assess these variables, future research could examine how individual differences contribute to phobic responses measured by the PSRS in Turkish samples. Exploring these relationships may provide deeper insights into fear susceptibility and enhance the scale’s clinical applicability.

Finally, the validation of the Turkish PSRS also provides an opportunity to advance both theoretical and cultural understanding of phobic responses within the Turkish context. The finding that the five-factor structure replicated in this sample supports theoretical models positing that specific phobias cluster into stable latent dimensions—such as animal, blood-injection-injury, situational, and harm-related fears—reflecting underlying biological, cognitive, and affective systems. Because the PSRS is grounded in cognitive-behavioral frameworks emphasizing threat appraisal, attentional bias, and catastrophic thinking, its multidimensional nature is well-suited to capturing these mechanisms. Integrating this theoretical structure with cultural considerations is particularly important in Türkiye, where collectivistic social norms, sensitivity to social evaluation, and heightened awareness of environmental threats (e.g., natural disasters) may shape the prominence or expression of certain fear domains, especially social and bodily-harm fears. Thus, the Turkish PSRS not only allows for reliable measurement but also opens a path for examining how culturally embedded beliefs, socialization practices, and environmental factors influence the organization of phobic responses. Future research that combines established theoretical models with culturally specific patterns will be critical for refining the conceptualization of phobic fear in Turkish populations and determining whether these dimensions manifest similarly or diverge meaningfully from patterns observed in Western samples. Given the potential influence of cultural norms and socialization practices on fear expression, future studies would benefit from testing the measurement invariance of the PSRS across Turkish and non-Turkish samples. Establishing configural, metric, and scalar invariance would clarify whether the scale’s latent structure functions equivalently across cultures, thereby enabling meaningful cross-cultural comparisons of phobic responses.

Despite its strengths, this study has certain limitations. The sample was limited to university students, predominantly female, and data were collected through convenience sampling, which may restrict the generalizability of the findings to broader and more diverse populations. Additionally, the reliance on self-report measures could introduce biases related to social desirability or response tendencies. Furthermore, while online data collection and self-report measures allow for greater reach, they may also raise concerns about data reliability. Future studies should aim to validate the Turkish PSRS in clinical samples and utilize multi-method assessment approaches, including behavioral and physiological measures, to further establish its validity evidence based on the internal structure. Moreover, future research should investigate the classification and underlying mechanisms of phobic responses in Turkish populations, particularly by incorporating clinical samples and examining personality-related influences. By doing so, a more comprehensive understanding of phobia and its assessment may be achieved, further enhancing the PSRS’s applicability in both research and clinical settings.

## Conclusions

The findings of this study provide strong evidence for the reliability and validity of the Turkish adaptation of the Phobic Stimuli Response Scales (PSRS) in a non-clinical sample. The scale demonstrated a stable factor structure, strong convergent and discriminant validity, and high internal consistency, confirming its effectiveness in assessing phobic responses across various fear domains. Given the limited availability of measurement tools developed or adapted for assessing fears and specific phobias in Turkish samples, this study fills an important gap by providing a psychometrically sound instrument that can be used both in research and clinical practice.

Moreover, the PSRS stands out as a practical tool due to its concise structure, consisting of 46 items that allow for quick and easy administration. Since the items are formulated as complete sentences, they enhance readability and comprehension, enabling participants to provide more accurate responses on a Likert-type scale. This user-friendly format not only facilitates data collection but also increases the scale’s applicability in various settings.

By validating the Turkish version of the PSRS, we hope to encourage further research on specific phobias in Türkiye and contribute to the growing body of literature in this field. Future studies can build on these findings to explore phobic responses in diverse populations and expand the scope of psychological assessments available in Turkish-language contexts.

## Supplementary Information

Supplementary Material 1

## Data Availability

The datasets used and/or analyzed during the current study are available from the corresponding author on reasonable request.

## Abbreviations

PSRS: Phobic Stimuli Response Scales
DASS-21: Depression, Anxiety, Stress Scale
APA: American Psychological Association
DSM-IV-TR: Diagnostic and Statistical Manual of Mental Disorders, Fourth Edition (Text Revision)
APA: American Psychiatric Association
DSM-5: Diagnostic and Statistical Manual of Mental Disorders, Fifth Edition
ADIS-5: Anxiety and Related Disorders Interview Schedule for DSM-5
SCID: The Structured Clinical Interview for DSM-5
FSS-II: Fear Survey Schedule Second Edition
CFA: Confirmatory factor analysis
DWLS: Diagonally weighted least squares
CFI: Comparative fit index
TLI: Tucker-Lewis index
RMSEA: Root mean square error of approximation
SRMR: Standardized root mean square residual
ICC: Intraclass correlation coefficient
