# Supplementary material for: Validation of the Turkish version of the Phobic Stimuli Response Scales
Source: Psicol Reflex Crit. 2026 Jan 30;39:7. doi: 10.1186/s41155-026-00380-7 (PMC12950129; doi:10.1186/s41155-026-00380-7)
Supplement: Supplementary file 1 — Supplementary Material 1 [file 41155_2026_380_MOESM1_ESM.docx]

10/12/2025

Dear Dr. Luis Manuel Lozano,

We are pleased to submit the revised version of our manuscript entitled “Validation of the Turkish Version of the Phobic Stimuli Response Scales.” We would like to express our sincere appreciation to the reviewers for their constructive feedback. Their insightful comments have been invaluable in improving the clarity, organization, and overall quality of our work, and we are grateful for the time and attention they dedicated to our submission.

In response to their suggestions, we have carefully revised the manuscript. A detailed table summarizing the reviewers’ comments and our corresponding responses is provided below. All changes made in the manuscript are highlighted in red to facilitate the review process. In addition to the reviewers' suggestions, minor corrections and additions deemed necessary by the authors are also indicated in red in the revised manuscript.

We hope that these revisions adequately address the reviewers’ concerns and meet your expectations. We sincerely appreciate your time and consideration and look forward to your feedback.

Thank you for your attention and continued support.

| **#s** | **Reviewer’s Comments/Suggestions** | | **Authors’ Responses/Corrections/Explanations** | |
| --- | --- | --- | --- | --- |
| **Reviewer #1** | | | | |
|  | | This is a well-crafted and valuable manuscript that addresses an important gap in the Turkish psychological assessment literature. The authors have successfully adapted the Phobic Stimuli Response Scales (PSRS) and demonstrated robust psychometric properties in a large non-clinical sample. The study is methodologically sound, the statistical analyses are appropriate, and the writing is clear and of high academic standard. I support the publication of this article. However, I believe that the inclusion of a few additional elements would further enhance the study's clarity, relevance, and rigour. | | We would like to thank the reviewer for their feedback. Their valuable suggestions have helped make the article more comprehensible. |
| **1** | | **Major Suggestions:**  **Abstract**  I think that the verb “investigate” in the objective needs to replace by the verb “evaluate”, in addition to being what was done and to be consistent with what the authors say in the text, more specifically in line 76. Please, verify. | | In accordance with the reviewer’s suggestion, the verb “investigate” has been changed to “evaluate”. (See line 5).  The correction is included in the manuscript as follows:  This study aimed to adapt the Phobic Stimuli Response Scales (PSRS) into Turkish and evaluate its psychometric properties. |
| **2** | | **Introduction**  Lines 62–63: "...currently no comprehensive scale ..."? In the world or in Turkey? | | Thank you for your clarification. The relevant section has been detailed. Please see lines 70-126.  Also, please see lines 133-134: To the best of our knowledge, no such measurement tool currently exists in Turkish literature. |
| **3** | | **Methods**  **Participants**  Lines 93–98: Please, could you clarify that the age range 18–53? | | In response to the reviewer's question mark, participants were university students. However, as some students are attending their second university, the maximum age observed is 53. The corrected sentence in the manuscript is as follows (Line 146):  The study included 399 university students (308 females, 77.2%; 91 males, 22.8%) aged between 18 and 53 years (*M* = 21.80, *SD* = 3.93), with 94% of them being 26 or younger. |
| **4** | | **Procedure**  Lines 100: Please, include ethics approval ID. | | The ethics committee approval date and decision number have been added to the manuscript. The corrected sentence is as follows (Lines 185-186):  Following this process, data collection commenced after obtaining ethical approval from X University’s Ethics Committee (Date: 22 July 2022; Number: 124/52). |
| **5** | | Lines 124–135:  The translation-retroversion process needs more rigor. Clarify who did the back-translation and whether any expert panel was used to resolve discrepancies, namely a bilingual committee to ensure semantic and conceptual equivalence. There are some studies that explained this one, for example see <https://pubmed.ncbi.nlm.nih.gov/11124735/> | | Thank the reviewer for this valuable feedback. The translation process was clarified. The corrected paragraph is as follows (Lines 176-184):  Before conducting the study, permission was obtained from the original authors for the Turkish adaptation of the PSRS. After receiving the necessary permission, the translation process began. During the translation process, the authors followed the guidelines (e.g., ITC Guidelines for Translating and Adapting Tests, 2017) regarding scale adaptation processes. Firstly, the scale was translated into Turkish separately by the researchers, and then the consistency between the translations was examined to determine the final form of the Turkish scale. Following this, a back-translation of the Turkish scale into English was conducted by an independent field expert to ensure compatibility with the original version. Subsequently, the Turkish scale was applied to a pilot sample of five individuals to examine its comprehensibility. |
| **6** | | **Results**  The CFA indices are very well reported (Table 1), and test-retest reliability (Table 4) is excellent. But:  Line 197: Item 35 - Please, note its low factor loading (.34) and discuss whether it should be revised or dropped in future studies. | | The following sentence was added to the Results section in the manuscript (Lines 216-218): The fit indices indicated adequate support for the five-factor structure, with factor loadings ranging from .50 to .89, except for item 35 (*λ* = .34, though still significant).  Also, the following paragraph was added to the Discussion section in the manuscript (Lines 274-284): Although the loading remained statistically significant, its magnitude suggests that the item may not represent the underlying construct as strongly as the rest of the scale. Given that item 35 assesses social ease rather than a direct fear response, cultural interpretations of shyness, interpersonal comfort, or social desirability may influence how Turkish participants respond to this item. Future studies should therefore examine whether item 35 would benefit from revision to improve clarity or cultural relevance, or whether removing it altogether would enhance the scale’s factorial precision without compromising its theoretical scope. Replication in clinical samples and cognitive interviewing methods may also help clarify whether the item reflects a broader personality trait rather than a domain-specific phobic tendency. Nevertheless, the item was retained in the Turkish adaptation to maintain consistency with the original scale and to preserve the structural integrity of the full 46-item measure. |
| **7** | | **Discussion**  It is a good discussion, but I think that the article and the study would benefit if the authors expanded cultural commentary and add implications for Turkish populations. | | Thank you very much for this valuable feedback. The following paragraph was added to the Discussion section in the manuscript (Lines 327-348):  Finally, the validation of the Turkish PSRS also provides an opportunity to advance both theoretical and cultural understanding of phobic responses within the Turkish context. The finding that the five-factor structure replicated in this sample supports theoretical models positing that specific phobias cluster into stable latent dimensions—such as animal, blood-injection-injury, situational, and harm-related fears—reflecting underlying biological, cognitive, and affective systems. Because the PSRS is grounded in cognitive-behavioral frameworks emphasizing threat appraisal, attentional bias, and catastrophic thinking, its multidimensional nature is well-suited to capturing these mechanisms. Integrating this theoretical structure with cultural considerations is particularly important in Türkiye, where collectivistic social norms, sensitivity to social evaluation, and heightened awareness of environmental threats (e.g., natural disasters) may shape the prominence or expression of certain fear domains, especially social and bodily-harm fears. Thus, the Turkish PSRS not only allows for reliable measurement but also opens a path for examining how culturally embedded beliefs, socialization practices, and environmental factors influence the organization of phobic responses. Future research that combines established theoretical models with culturally specific patterns will be critical for refining the conceptualization of phobic fear in Turkish populations and determining whether these dimensions manifest similarly or diverge meaningfully from patterns observed in Western samples. Given the potential influence of cultural norms and socialization practices on fear expression, future studies would benefit from testing the measurement invariance of the PSRS across Turkish and non-Turkish samples. Establishing configural, metric, and scalar invariance would clarify whether the scale's latent structure functions equivalently across cultures, thereby enabling meaningful cross-cultural comparisons of phobic responses. |
| **8** | | Line 244-256: Although the authors acknowledge the limitations of their study—such as the reliance on a predominantly female, non-clinical university sample and the use of convenience sampling—it is important to emphasise that these constraints do not diminish the relevance and contribution of the current work. Despite the limited generalisability, the study provides a valuable and psychometrically robust instrument that fills a clear gap in the Turkish psychological assessment landscape. The current study offers an essential starting point for the use of the PSRS in both Turkish research and applied settings.  Note: If possible, include similar limitations have been reported in previous scale validation studies involving fear or phobia assessments | | Thank you for this valuable comment and feedback. Despite the limited generalizability, the study provides a valuable and psychometrically robust instrument that fills a clear gap in the Turkish psychological assessment landscape. The current study offers an essential starting point for the use of the PSRS in both Turkish research and applied settings. The strength of the adaptation was mentioned in the aforementioned paragraph (Lines 327-348) and the conclusion part (Lines 363-378). |
| **9** | | **Conclusions**  Line 260: Maybe is better to say, "discriminant validity" instead of "divergent validity"? | | Thank you for this clarification. The divergent validity was changed as discriminant validity in 7 different places in the manuscript (see lines 12, 229, 231, 286, 293, 365, 563) |
| **Reviewer #2** | | | | |
|  | | Thank you very much for the opportunity to review this manuscript. The study aims to validate a Phobic Stimuli Response Scale in a university population in Turkey. The topic is relevant and potentially contributes valuable insights to the field of psychological assessment and phobia research. While the methodological approach is generally well-developed, the manuscript requires several improvements in its theoretical foundation, structure, and reporting of statistical analyses before it can be considered for publication. | | We would like to thank the reviewer for their kind comments and suggestions. |
| **1** | | **Abstract**  Please include statistical data to support the results mentioned in the abstract. This will enhance the clarity and strength of the findings presented. | | In accordance with APA 7 formatting, it is not recommended to include too many numerical/statistical values in the abstract section; therefore, the authors have followed this approach in the first draft of the manuscript. Although the reviewer's suggestion is highly valuable, it is not clear which values should be included in which section. Nevertheless, the authors have made the following corrections in the abstract (see Lines 3-17):  Background: The current literature lacks measurement tools that cover all subtypes of specific phobias. Objective: This study aimed to adapt the Phobic Stimuli Response Scales (PSRS) into Turkish and evaluate its psychometric properties. Methods: 399 university students (308 females, 77.2%; 91 males, 22.8%) aged between 18 and 53 years (*M* = 21.80, *SD* = 3.93) filled out the PSRS, Fear Survey Schedule-II, and Depression, Anxiety, Stress Scale (DASS-21) online. 108 participants took part in the test-retest study 15 days after the first application. Results: According to confirmatory factor analysis, model fit indices (CFI = .97, TLI = .97, RMSEA = .07, and SRMR = .07) showed that the Turkish form of the scale consisted of 46 items and five factors (Blood-Injection, Bodily Harm, Social, Animal, and Physical Confinement) as in the original. Its correlations with other scales showed its convergent and discriminant validity. The reliability analyses indicate that the scale demonstrates internal consistency (*α =* .78 for Bodily Harm, .85 for Physical Confinement, .88 for Social, .86 for Animal, .90 for Blood-Injection, and .94 for the total score) and test-retest reliability. Conclusions: The findings suggest that the Turkish PSRS is a reliable and valid instrument for assessing responses to various fears in research and practical applications.  **Keywords:** Fear, Phobias, Phobic stimuli, Validity, Reliability |
| **2** | | **Introduction**  The theoretical framework is outdated and underdeveloped. Please update this section by incorporating recent and relevant literature. | | **We thank the reviewer for their suggestion. The text, as it appeared in the first draft, has been revised in the revision file. For the revised text, please see Lines 49-126.**  Specific phobias are the most prevalent anxiety disorder and the third most common mental disorder in the general population. Epidemiological studies indicate that approximately 12.5% of individuals will experience a specific phobia at some point in their lifetime (Kessler et al., 2005).  Despite the high prevalence of specific phobias, the number of measurement tools assessing this condition remains limited in the relevant literature. For instance, the Specific Phobia Questionnaire (Fairbrother & Antony, 2012; Ovanessian et al., 2019), developed in accordance with DSM-5 criteria, evaluates fear responses to 45 different situations and the extent to which these fears interfere with daily life. However, a Turkish adaptation of this scale has not yet been conducted. Additionally, the DSM-5 Severity Measure for Specific Phobia Scale (Adult) is a 10-item self-report measure assessing the frequency of anxiety, fear, and avoidance behaviors in response to five different situations or objects. The scale employs a five-point Likert-type format, where each symptom is rated based on its frequency over the past seven days, allowing individuals to indicate the severity of their phobic responses (Craske et al., 2013; Turkish adaptation by Öztekin et al., 2017). Finally, existing phobia scales in the Turkish literature have primarily focused on specific fear domains, such as agoraphobia (e.g., Aydın et al., 2017), blood-injury phobia (e.g., Ak et al., 2014), and social phobia (e.g., Cengiz et al., 2018), each assessed through separate instruments. However, there is currently no comprehensive scale that encompasses all specific phobia types within a single measure.  To address the measurement tool gap in the literature, Cutshall and Watson (2004) developed a scale assessing the cognitive and emotional aspects of fear responses. They intentionally avoided labeling it as a “phobia” since their data were collected from an undergraduate sample. However, they preferred to name the scale the Phobic Stimuli Response Scales (PSRS), recognizing that nonclinical samples still experience fear and that such data can provide valuable insights for clinical populations (Cutshall & Watson, 2004). Additionally, they used full sentences to describe emotional responses to a given stimulus, as explicit wording helps minimize individual differences in item interpretation and provides a clearer understanding of various emotional reactions (Cutshall & Watson, 2004). Therefore, they developed the PSRS, a 46-item scale comprising five sub-dimensions—Blood-Injection, Bodily Harm, Social, Animal, and Physical Confinement—measured on a four-point Likert-type scale ranging from 1 (Strongly disagree) to 4 (Strongly agree). |
| **3** | | The references cited are scarce and do not sufficiently support the rationale for the study. A more robust theoretical grounding is necessary to justify the scale's validation. | | In accordance with the reviewer’s suggestion, as stated in the previous response (#2), current literature and theoretical bases have been added to the introduction (Lines 49-126). More recent studies have been added to the References section. |
| **4** | | **Method**  **Participants:**  Please specify the academic disciplines or majors of the university students participating in the study. | | In the original scale development article (Cutshall & Watson, 2004), the authors did not include information about the university students' faculties and departments. In the adaptation study, all stages in the original article were attempted to be followed, and this information was not requested from the participants. |
| **5** | | **Instruments:**  Replace the heading Measures with Instruments to adhere to standard APA terminology. | | Thank the reviewer for this clarification. It was corrected as Instruments (Line 150). |
| **6** | | Ensure consistency in formatting: some instrument names are italicized while others are not. Please revise for coherence and adherence to journal style guidelines. | | The Phobic Stimuli Response Scales (PSRS) subheading was italicized. (Line 157) |
| **7** | | **Procedure:**  The current description is insufficient. This section should clearly explain how the sample was collected, including recruitment methods, inclusion/exclusion criteria, and ethical procedures. | | Thank you for this suggestion. The added sentence is as follows (Lines 188-190):  The researchers disseminated the study poster and participation link via class groups and personal social media platforms and encouraged university students who completed the scales to forward the link to another participant. |
| **8** | | **Scale Adaptation:**  The process of adapting the scale is briefly described and located incorrectly. I recommend relocating this content to the Instruments section, specifically under the description of that instrument. | | Basic information regarding the scale is provided under the Instruments heading. However, as far as we know, and as seen in other adaptation articles in “Psychology: Research and Review”, translation and back-translation processes, along with pilot applications, constitute components of the research process and are therefore presented under the heading of Procedure. For this reason, we regretfully have to decline the reviewer’s suggestion. |
| **9** | | **Data Analysis:**  This section is missing entirely and must be included. Clearly describe all statistical procedures used in the validation process, including reliability and validity analyses. | | Thank you for your attention and suggestions. The data analysis section and the following paragraph were added to the manuscript (Lines 195-204):  **Data Analysis**  All statistical analyses were performed using JASP software (JASP Team, 2023). To provide validity evidence based on the internal structure, a confirmatory factor analysis was conducted. Convergent (the relationship between the PSRS total score and FSS-II total score) and discriminant (the relationship of DASS-21 subscales with the PSRS subscales and the PSRS total score) validity, as well as test-retest reliability, were examined using Pearson’s product-moment correlation analysis. Additionally, the intraclass correlation coefficient was calculated to further evaluate test-retest reliability. For reliability assessment, Cronbach’s alpha coefficients, along with corrected item–total and item–subscale correlations, were computed. Finally, descriptive statistics, including means and standard deviations, were calculated to examine item-level characteristics. |
| **10** | | **Results**  Do not refer to "construct validity." Instead, use the term "validity evidence based on internal structure," in line with current psychometric standards (e.g., AERA, APA, NCME Standards). | | The term construct validity was changed to validity evidence based on the internal structure in 2 different places in the manuscript (please see lines 210, 356, 357). |
| **11** | | Please include a table showing descriptive statistics for all items, as well as corrected item-total correlations (item discrimination indices). | | Item descriptive statistics (mean and standard deviation for all items) and corrected item-subscale and corrected item-total correlations were added to Table 2 as additional columns. Table 2 was reorganized. |
| **12** | | Include a figure depicting the Confirmatory Factor Analysis (CFA) model, and specify whether modification indices were used and applied. | | Modification indices were not used in the CFA. It was added to the manuscript as follows (Line 216):  No modification was used in the CFA.  Figure 1, which shows the path diagram of the CFA, was added to the manuscript. |
| **13** | | For the test-retest reliability analysis, please report the intraclass correlation coefficient (ICC), which is more appropriate than other types of correlation coefficients. | | The intraclass correlation coefficients (ICCs) for the subscales and the total PSRS were added to Table 4 as an additional column and to the related text in the manuscript (please see Table 4 and lines 201, 248, 313-314, 396, 567). |
| **14** | | **Discussion**  The discussion is generally well-structured and clearly written. However, it lacks integration with a comprehensive theoretical framework, which is also missing from the introduction. Revisiting and expanding the theoretical discussion will strengthen the interpretation of the findings. | | Thank you very much for this valuable feedback. The following paragraph was added to the Discussion section in the manuscript. (Lines 327-348)  Finally, the validation of the Turkish PSRS also provides an opportunity to advance both theoretical and cultural understanding of phobic responses within the Turkish context. The finding that the five-factor structure replicated in this sample supports theoretical models positing that specific phobias cluster into stable latent dimensions—such as animal, blood-injection-injury, situational, and harm-related fears—reflecting underlying biological, cognitive, and affective systems. Because the PSRS is grounded in cognitive-behavioral frameworks emphasizing threat appraisal, attentional bias, and catastrophic thinking, its multidimensional nature is well-suited to capturing these mechanisms. Integrating this theoretical structure with cultural considerations is particularly important in Türkiye, where collectivistic social norms, sensitivity to social evaluation, and heightened awareness of environmental threats (e.g., natural disasters) may shape the prominence or expression of certain fear domains, especially social and bodily-harm fears. Thus, the Turkish PSRS not only allows for reliable measurement but also opens a path for examining how culturally embedded beliefs, socialization practices, and environmental factors influence the organization of phobic responses. Future research that combines established theoretical models with culturally specific patterns will be critical for refining the conceptualization of phobic fear in Turkish populations and determining whether these dimensions manifest similarly or diverge meaningfully from patterns observed in Western samples. Given the potential influence of cultural norms and socialization practices on fear expression, future studies would benefit from testing the measurement invariance of the PSRS across Turkish and non-Turkish samples. Establishing configural, metric, and scalar invariance would clarify whether the scale's latent structure functions equivalently across cultures, thereby enabling meaningful cross-cultural comparisons of phobic responses. |
| **15** | | **Conclusion**  The manuscript addresses an important topic and shows potential, but significant revisions are required to meet publication standards. I encourage the authors to revise the manuscript thoroughly, especially by reinforcing the theoretical background, ensuring methodological clarity, and improving the reporting of statistical analyses. | | In line with the reviewer’s suggestions, the theoretical background has been strengthened, methodological clarity has been ensured, and the reporting of statistical analyses has been improved. We thank you for your suggestions, which have made the article more comprehensible. |
